# Effects of Music Training on Social, Behavioral, and Academic Skills of Children with Cochlear Implants from the School Teacher's Perspective

**DOI:** 10.1055/s-0045-1809646

**Published:** 2025-08-20

**Authors:** Paula Martins-Said, Kátia de Freitas Alvarenga, Dagma Venturini Marques Abramides

**Affiliations:** 1 Cochlear Implant Division, Hospital de Reabilitação de Anomalias Craniofaciais, Universidade de São Paulo, Bauru, SP, Brazil; 2 Audiology Program, Department of Speech-Language Pathology, Faculdade de Odontologia de Bauru, Universidade de São Paulo, Bauru, SP, Brazil

**Keywords:** music, deafness, cochlear implantation, social skills

## Abstract

**Introduction:**

Music has been recognized as a therapeutic tool in auditory rehabilitation, promoting essential social and behavioral development in children with cochlear implants (CIs), as identified by the World Health Organization (WHO). Additionally, there is a lack of studies on the development of these skills in this context.

**Objective:**

To investigate the effect of music training on the development of social, behavioral, and academic skills in children with CIs from the schoolteacher's perspective.

**Methods:**

The present is an experimental study involving 10 children with CIs, aged between 6 and 10 years, who composed the experimental group. They had a semester of music training and were assessed through the Social Skills Rating System (SSRS) questionnaire at 4 different moments: 2 months before the beginning of the music training, at the beginning of the music training (first week of class), during the music training (3 months after the beginning), and at the end of the music training (6 months after the beginning). To compare the multiple SSRS assessments, we used the repeated measures analysis of variance (ANOVA) test and the Tukey test ( *p* ≤ 0.05).

**Results:**

The results showed a statistically significant improvement in social skills in terms of assertiveness, social resourcefulness, self-control, affectivity, cooperation, externalizing behavior problems, and academic competence.

**Conclusion:**

From the schoolteacher's perspective CI-using children who had music training experienced an improvement in their social and academic skills and behavioral problems.

## Introduction

A growing number of experimental studies use music as an intervention procedure to analyze its benefits in cochlear implant (CI) users. The results have demonstrated that musical interventions can be an effective strategy to help develop auditory skills for the perception of speech and oral language. However, the length of sensory deprivation and the absence of binaurality can hinder such development. Binaural sound information processing is essential for individuals without hearing loss and for CI users, 5 and it is related to more complex auditory abilities such as speech perception in noisy environments and music perception.

The specific literature shows that CI users exposed to music training experience an improvement on their linguistic skills, auditory perception, 2 and speech production. 1 6 7 Moreover, studies have demonstrated a tendency toward an increase in the habit of listening, enjoying, and engaging with music after CI surgery, 8 9 associating these practices with their self-perceived quality of life. 10

The similar hierarchical structures in the process of learning music and language suggest a connection between them in terms of higher cognitive functions and subsystems, such as memory, attention, and categorization. 11 This assumption has been reinforced by studies that demonstrated 12 13 14 that musical learning favored brain plasticity and expanded connections among neurons, including mirror neurons, which are recruited through imitation in both action and observation of activity. These are neurons responsible for building productive and healthy interpersonal relationships 12 13 and learning, 14 which are essential to child neurodevelopment.

Additionally, attention has been given to understanding emotional processing in CI users, since important acoustic cues are missed not only by artificial electrical stimulation but also due to auditory neural changes resulting from the period of sensory deprivation. Ascribing meaning, whether to a song or the speaker's voice, reflects the emotion that the individual experiences through hearing and, consequently, their emotional response to the situation. 15 16

Children with severe or profound hearing loss have limited or no access to spoken language, which hinders their development of socioemotional skills 17 18 and increases their likelihood of developing behavioral problems, 19 20 as language enables emotional self-regulation and sociocognitive skills. 21 Thus, social skills are essential for full child development. 22

Social skills are essential social behaviors to build healthy and productive interpersonal relationships. 23 They encompass academic competence and behavioral problems, 24 25 26 divided into externalizing behaviors (such as inattention, aggressiveness, and hyperactivity), 27 and internalizing ones, such as depression and anxiety. 28 Said and Abramides 23 and Said et al. 29 pointed out that aspects of the musical learning process were responsible for promoting social skills, academic competence, and behavioral problems in typically-developing children.

Music training can enhance children's social skills by engaging them in active, structured learning experiences that include musical activities such as playing instruments and singing. These experiences not only foster the development of musical abilities but also activate physiological, cognitive, and emotional processes that influence non-musical outcomes, such as social skills. 30 Thus, research suggests that active music training 23 29 31 32 promotes interpersonal communication, empathy, and collaboration. These benefits are linked to the way music taps into both cognitive and emotional processes that are essential for social functioning.

Building on these connections, the seven social functions of music proposed by Koelsch 33 provide a framework to understand how music contributes to social and emotional development. These functions—social contact, social cognition, copathy (empathy), communication, coordination of actions, cooperation, and social cohesion—highlight music's integral role in fostering interpersonal relationships and group cohesion. For children, engaging in music can serve as a medium for emotional expression and social interaction, 31 32 supporting the development of key socioemotional skills, including those using CIs.

Literature reviews 8 9 34 35 have shown that when CI-using children with severe or profound sensorineural hearing loss are exposed to early intervention combined with musical activities, they experience an improvement on their development of auditory, social, speech, and language skills. However, there were few studies 10 36 37 in the researched literature that evaluated or merely reported the effects of music training on socioemotional skills, and no studies were found on academic skills or behavioral problems in children using bilateral CIs.

The present study hypothesized that music training based on the Music Learning Theory (MLT) 38 in children using bilateral CIs will influence their development of social, behavioral, and academic skills, positively reflecting on academic classroom activities.

In this sense, the objective of the current study was to investigate the effect of music training on the set of social, behavioral, and academic skills in children with hearing loss who use bilateral CIs, from the perspective of regular schoolteachers.

## Methods

This controlled, longitudinal, non-randomized, blind clinical study is an integral part of the research group named “Centro de Pesquisas Audiológicas - CNPq”, linked to the Speech-Language-Hearing Postgraduate Program of the Dental School of Bauru, University of São Paulo (FOB/USP), Bauru campus, in partnership with the Hospital for Rehabilitation of Craniofacial Anomalies (HRAC) and the Bravo Music Academy, based in Bauru.

This study was approved by the Research Ethics Committee of the Dental School of Bauru FOB/USP, protocol no. 2.820.891.

### Selection of the Sample

Children enrolled in the Specialized Center for Hearing Development (CEDAU) of the Hospital for Craniofacial Anomalies (HRAC/USP) were invited to participate in this study, according to the following inclusion criteria: 1) age from 6 to 10 years, as it is pointed out as an excellent stage of life to acquire and develop academic skills and consolidate the set of social skills. Socially, children transition from family-centered interactions to forming peer relationships and engaging in group dynamics. Academically, foundational skills like literacy and numeracy are consolidated, along with higher-order cognitive skills, such as reasoning. Academic skills refer to abilities such as attention, task persistence, self-regulation, and communication, while consolidating social skills involve refining behaviors like cooperation, empathy, and conflict resolution. These milestones make this age group ideal to study interventions such as music training, which may enhance these skills; 23 2) both sexes; 3) bilateral severe or profound sensorineural hearing loss; 4) bilateral CI users; 5) no complaints related to neurological and psychiatric problems; and 6) intellectual level within normal parameters, assessed through the Raven's Colored Progressive Matrices (CPM) 39 applied by a psychologist external to the research. This instrument is a nonverbal test that assesses general intelligence regarding analogical reasoning aptitude, that is, the child's ability to deduce relationships involving objects or elements, which is one of the main components of general intelligence (g factor).

The exclusion criteria were as follows: children with bilateral severe or profound postlingual sensorineural hearing loss; unilateral CI users; non-residents of the city where the study was performed; children who had already had music classes; and those who did not attend the proposed music training effectively.

### Participants

Twenty randomly divided children who met the inclusion criteria were divided into the experimental group (EG) and the control group (CG). During data collection, 70% of the children in the CG dropped out, because they were waiting for the intervention and due to factors external to the study, such as the beginning of the coronavirus disease 2019 (COVID-19) pandemic. The remaining 30% of the CG participants were reallocated to another study due to the small number of participants left in the group.

Thus, the sample consisted of 10 children with profound hearing loss, users of bilateral CI, oralized, with mean time of CI use of 3.7 years, and enrolled in elementary school. There were 6 girls and 4 boys, with a mean age of 6.9 years, with a mean age at implantation of 3.2 years, with low ( *n* = 7; 70%) and very low socioeconomic status ( *n* = 3; 30%), public school students ( *n* = 8; 80%) and with congenital hearing loss ( *n* = 10; 100%) . All participants attended all music training sessions.

### Instruments

The current study used the teacher form of the Social Skills Rating System–Brazilian version (SSRS-BR). This scale was originally produced in the United States, 40 and it was validated for the Brazilian context by Freitas and Del Prette, 41 with psychometric qualities verified in terms of internal consistency and temporal stability for preschoolers and children in the first to fourth grades. It contains three subscales – social skills, behavior problems, and academic competence –, totaling 38 items for analysis. The factors in the social skills subscale are Global score, F1–responsibility, F2–self-control, F3–assertiveness/social resourcefulness, and F4–affection/cooperation. The factors in the behavioral problems subscale are Global score, F1–externalizing, F2–hyperactivity, and F3–internalizing. And the academic competence subscale is composed of the Global Score. The results are based on the scores correlated with the percentile scale provided in the application manual, which classifies social skills as below the lower average, within the lower average, good, elaborate, and highly-elaborate; behavior problems as very low, low, average, upper-average, and above upper average; and academic competence as below the lower average, within the lower average, in the median, high, and very high. No other studies have used the SSRS with pediatric CI users.

### Procedures

The teachers answered the self-administered teacher form of the SSRS-BR. 41 The data were investigated and interpreted by a psychologist external to the research with experience in using the questionnaire.

The teacher form of the SSRS-BR was presented to teachers at 4 different moments: M1–2 months before the beginning of music training; M2–at the beginning of music training (first week of class); M3–during music training (3 months after the beginning); and M4–at the end of music training (6 months after the beginning).

### Intervention Procedure

The MLT, a musical learning approach for children, 38 was performed by a music educator specializing in the method who was external to the research.

The MLT is a music teaching and learning theory that focuses not only on the teaching of music but also on the process through which it is learned, aiming to achieve musical understanding, which the author Edwin Gordon 38 terms *audiation* . The use of the MLT as an intervention procedure is justified by the fact that this teaching and learning theory is structured through sequenced learning activities. These activities use musical patterns, which are basic units with musical meaning that can be compared with words, making music learning similar to speech learning, with emphasis on body movement associated with musical practice.

**Fig. 1:**
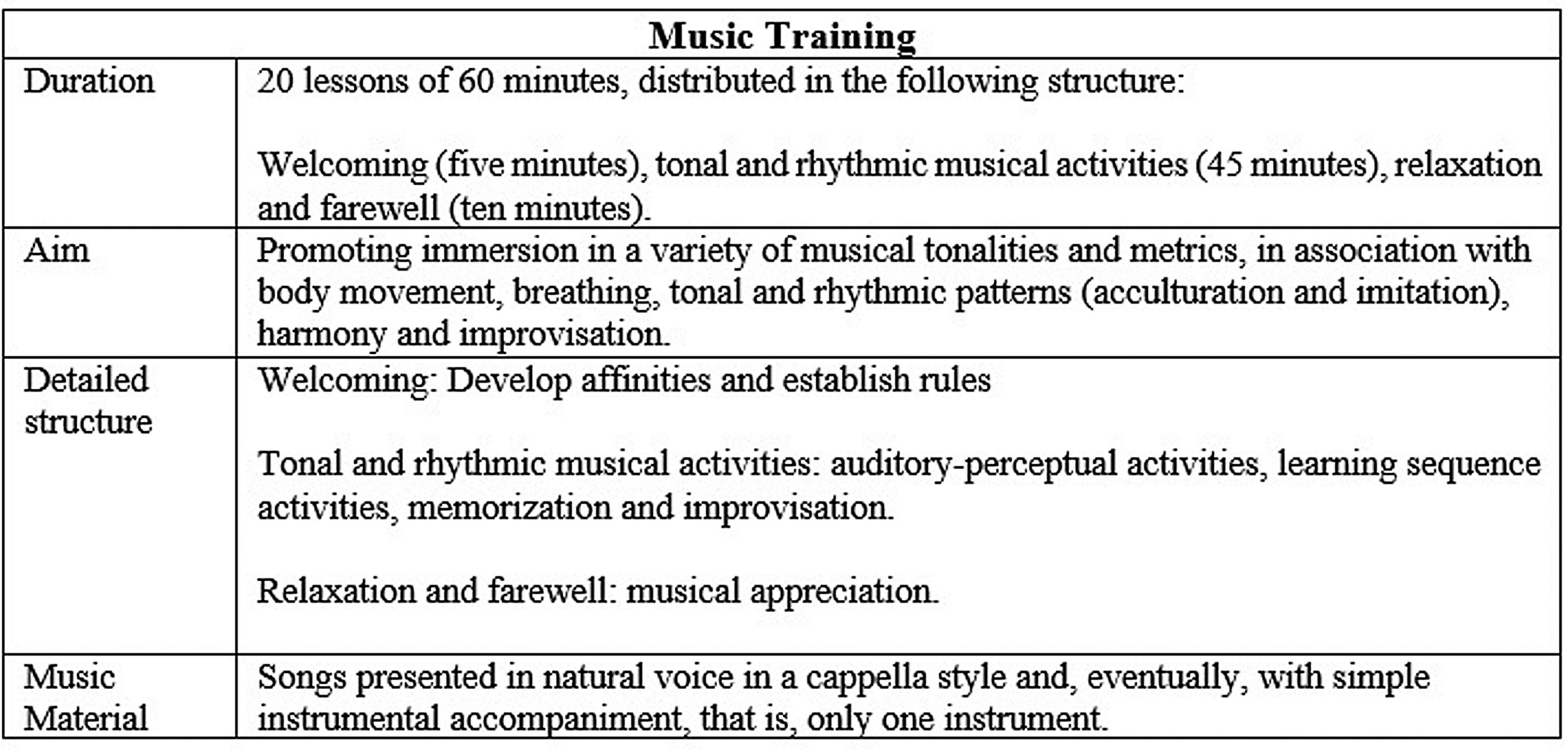
Details of the music training.

The MLT activities start with sound material familiar to the child, and the classes are structured around the use of the singing voice. The music training lasted 60 minutes, and it was divided into playful and dynamic musical activities. Weekly classes were held throughout the semester, totaling 20 classes. The classes had a collective format, with the 10 children in the EG doing the activities together belonging to the same class. Fig. 1 presents musical training details.

In the MLT framework, each stage serves a specific purpose to support the child's musical development:

*Welcoming* – this stage focuses on creating a welcoming and supportive environment. It helps develop affinities among participants and establishes clear rules and expectations for the session, fostering a sense of belonging and structure.

*Tonal and Rhythmic Musical Activities* – this is the core of the session, involving auditory-perceptual activities designed to enhance listening skills and musical understanding. It includes learning sequence activities, in which children are introduced to tonal and rhythmic patterns, as well as memorization, in which they internalize musical material, and improvisation, which enables them to explore and apply their musical knowledge creatively. The use of group singing is emphasized as a central activity, enabling children to engage with tonal and rhythmic patterns collectively, reinforcing their musical perception and fostering group cohesion.

*Relaxation and Farewell* – this stage emphasizes musical appreciation, giving children the opportunity to reflect on the musical experiences of the session in a calm and enjoyable way. It helps close the session on a positive note, reinforcing the learning and emotional connection with music.

**Appendix 1** presents a lesson example.

### Metehod Used or Result Analysis

The data were analyzed descriptively and inferentially, using the IBM SPSS Statistics for Windows (IBM Corp.), version 25.0, and the Sigmaplot (Grafiti LLC) software, version 12.0. Ordinal quantitative and qualitative variables were expressed using measures of variability (standard deviation), central tendency (mean and median), and position (minimum, maximum, and quartiles one and three) of the scores in each factor evaluated. Preliminarily, the normality of the data was determined using the Shapiro-Wilk test, with *p* ≤ 0.05. To compare the multiple analyses of the EG, we used repeated measures analysis of variance (ANOVA) and the Tukey test (post hoc), which indicates at which moment there was a statistical difference (M1, M2, M3, and M4). The significance level was set at 5% ( *p* ≤ 0.05) in all inferential analyses.

The qualitative analysis used the percentile scale as proposed in the instrument, with social skills scored as follows: 1 to 25–below the lower average; 26 to 35–within the lower average; 36 to 65–good; 66 to 75–elaborate; and 76 to 100–highly elaborate. The behavior problems were scored as: 1 to 25–very low; 26 to 35–low; 36 to 65–average; 66 to 75–upper average; and 76 to 100–above the upper average. Finally, academic competence was scored as: 1 to 25–below the lower average; 26 to 35–within the lower average; 36 to 65–in the median; 66 to 75–high; and 76 to 100–very high.

## Results

Table 1 shows the performance on the SSRS-BR in factors that encompass social skills in M1, M2, M3, and M4, as well as a comparative score analysis. The results indicated that the global score increased from 29.80 (M1) to 38.00 (M4) ( *p* = 0.001; post hoc: M1 > M4; M1 > M3); the percentiles improved from 35 (M1) to 75 (M4). Responsibility (F1) increased from 9.00 (M1) to 10.90 (M4), but this was not significant ( *p* = 0.071). The percentiles remained stable (from 30 in M1 to 40 in M4). Self-Control (F2) increased from 9.20 (M1) to 12.40 (M4) ( *p* = 0.001; post hoc: M1 > M4; M2 > M4; and M3 > M4); The percentiles increased from 25 (M1) to 55 (M4). Assertiveness/Social resourcefulness (F3) increased from 7.30 (M1) to 9.10 (M4) ( *p* = 0.026; post hoc: M1 > M4); The percentiles increased from 55 (M1) to 85 (M4). And affection/cooperation (F4) increased from 4.40 (M1) to 5.70 (M4) ( *p* = 0.003; post hoc: M1 > M4); the percentiles rose from 40 (M1) to 65 (M4).

**Table 1:** Performance in the factors that encompass social skills in the four assessment moments (M1, M2, M3, and M4) and comparative analysis of the scores per assessment moment

SSRS-BR (teacher form)	M1		M2		M3		M4		*p*	Post hoc
	Mean	Standard deviation	Mean	Standard deviation	Mean	Standard deviation	Mean	Standard deviation		
**Global score**	29.80	± 10.76	32.80	± 11.75	35.00	± 12.49	38.00	± 11.82	0.001*	M1 > M4 = 0.001;* M1 > M3 = 0.010;* M2 > M4 = 0.001*
***Percentile***	***35***		***45***		***60***		***75***			
**F1–responsibility**	9.00	± 2.75	10.20	± 2.39	10.00	± 2.94	10.90	± 2.28	0.071	
***Percentile***	***30***		***35***		***40***		***40***			
**F2–self-control**	9.20	± 2.94	10.60	± 3.10	10.60	± 3.37	12.40	± 2.76	0.001*	M1 > M4 = 0.001;* M2 > M4 = 0.025;* M3 > M4 = 0.025*
***Percentile***	***25***		***35***		***40***		***55***			
**F3–assertiveness/social resourcefulness**	7.30	± 2.11	7.90	± 1.97	8.40	± 2.22	9.10	± 1.60	0.026*	M1 > M4 = 0.019*
***Percentile***	***55***		***60***		***75***		***85***			
**F4–affection/ cooperation**	4.40	± 1.58	4.40	± 1.58	5.40	± 1.58	5.70	± 1.58	0.003*	M1 > M4 = 0.013*
***Percentile***	***40***		***40***		***60***		***65***			

Abbreviation: SSRS-BR, Social Skills Rating System–Brazilian version.

Note: *Statistically significant.

Table 2 shows the performance in factors that encompass the frequency of problematic behaviors in M1, M2, M3, and M4, as well as a comparative score analysis. The results indicated that the global score decreased from 4.30 (M1) to 1.50 (M4) ( *p* = 0.003; post hoc: M1 > M3; M1 > M4); the percentiles dropped from 50 (M1) to 20 (M4). Externalizing behaviors (F1) decreased from 1.30 (M1) to 0.50 (M1), which was not significant ( *p* = 0.280); the percentiles dropped from 45 (M1) to 20 (M4). Hyperactivity (F2) decreased from 2.70 (M1) to 1.20 (M4) ( *p* = 0.046; post hoc: M1 > M4); the percentiles decreased from 65 (M1) to 10 (M4). And internalizing behaviors (F3) increased from 0.64 (M1) to 0.91 (M4), which was not significant ( *p* = 0.770); the percentiles remained stable (from 25 in M1 to 35 in M4).

**Table 2:** Frequency of factors that encompass problematic behaviors in the four assessment moments (M1, M2, M3, and M4) and comparative analysis of the scores per assessment moment

SSRS-BR (teacher form)	M1		M2		M3		M4		*p*	Post hoc
	Mean	Standard deviation	Mean	Standard deviation	Mean	Standard deviation	Mean	Standard deviation		
**Global score**	4.30	± 2.98	3.30	± 2.26	2.30	± 1.70	1.50	± 1.43	0.003*	M1 > M3 = 0.038;* M1 > M4 = 0.002*
***Percentile***	***50***		***40***		***30***		***20***			
**F1–externalizing**	1.30	± 1.70	0.90	± 0.99	0.60	± 1.08	0.50	± 1.27	0.280	
***Percentile***	***45***		***40***		***30***		***20***			
**F2–hyperactivity**	2.70	± 1.77	1.90	± 1.52	1.60	± 1.43	1.20	± 1.40	0.046*	M1 > M4 = 0.034*
***Percentile***	***65***		***50***		***25***		***10***			
**F3–internalizing**	0.64	± 0.67	1.00	± 1.10	0.91	± 0.38	0.91	± 0.58	0.770	
***Percentile***	***25***		***40***		***35***		***35***			

Abbreviation: SSRS-BR, Social Skills Rating System–Brazilian version.

Note: *Statistically significant.

Table 3 shows the performance in factors that encompass academic skills in M1, M2, M3, and M4, as well as a score analysis. The results indicated that the global score increased from 27.46 (M1) to 32.55 (M4) ( *p* = 0.001; post hoc: M1 > M4; M1 > M3; and M2 > M4); the percentiles improved from 20 (M1) to 40 (M4).

**Table 3:** Academic competence in the four assessment moments (M1, M2, M3, and M4) and comparative analysis of the scores obtained per assessment moment

SSRS-BR (teacher form)	M1		M2		M3		M4		*p*	Post hoc
	Mean	Standard deviation	Mean	Standard deviation	Mean	Standard deviation	Mean	Standard deviation		
**Global score**	27.46	± 14.25	28.36	± 14.13	30.73	± 14.18	32.55	± 14.37	0.001*	M1 > M3 = 0.017;* M1 > M4 = 0.001;* M2 > M4 = 0.002*
***Percentile***	***20***		***30***		***35***		***40***			

Abbreviation: SSRS-BR, Social Skills Rating System–Brazilian version.

Note: *Statistically significant.

## Discussion

The development of musical perception in CI-using children has been studied in recent years, as it is directly linked to auditory processing and auditory skills acquired throughout a person's life.

Musical aptitude is an innate competence, but it is affected by the quality of the environment to which the child belongs, the richer it is musically, the more favorable it is for improving more specific auditory skills. 38

The data found in the present study demonstrated that, in the teacher's view, music training promoted significant changes in the attitudes of CI-using children in the classroom. This finding is in line with the MLT, which, as an intervention procedure, covers the basic elements of music, but also playfully addresses sensory, cognitive, perceptual, motor, social, and language factors.

Table 1 points out significant differences in the teachers' views regarding the improvement in the set of social skills, in terms of the global score, self-control, assertiveness/social resourcefulness, and affection/cooperation at the end of the music training – except for F1 (responsibility). Thus, the analysis of the instrument's percentile in the four assessment moments demonstrates that CI-using children started the study with a set of assertiveness/resourcefulness and affection/cooperation expected for their age – that is, with balanced interpersonal behaviors for coexistence in society. At the end of the training, there was significant improvement in these factors, as they began to demonstrate highly satisfactory and skillful behaviors, according to the teacher. In this context, this draws attention to the impact of music training on self-control, in which the behavior of CI-using children was below average, with an index considered critical for social and academic adjustment, and, at the end, they began to behave as expected for the age group ( Table 1 ). Such an evolution in the set of social skills is justified by the learning process resulting from the active music training structure, which establishes their interaction and communication with the teacher and peers and requires discipline, attentional focus, and the ability to follow rules. 23 29 30 31 32

Music training is described as an activity that triggers higher cognitive functions, such as the attention control subsystem, which helps develop self-control, responsibility, affection, and cooperation. Consequently, the child maintains concentration on a given task and ignores distracting factors, favoring the classes of social skills investigated, 11 additionally improving their quality of life. 10

In the analysis of behavior problems, we compared assessment moments ( Table 2 ) and found a result similar to that described previously, with a significant decrease in the frequency of hyperactive behavior problems and global score after music training – except for externalizing behaviors. As for internalizing behaviors, the results did not have a consistent pattern, which may reflect the teacher's difficulty in analyzing their aspects, such as anxiety, depression, and social phobia. The analysis of the percentiles shows that, for the global score and hyperactivity, CI-using children started with behaviors within the average, with a balance between interpersonal resources and deficits, and later achieved a very low set of behavior problems, indicative of highly satisfactory interpersonal resources ( Table 2 ).

The results are consistent with those of studies 24 25 that show that social skills prevent the development of problematic behaviors. Group practices in music training have a high socialization power and predispose the individual to lose selfishness and individualism 23 and interact with the teacher, considered in the literature as the main MLT conduction axis. 38

The learning process, including musical learning, stimulates the child's multiple connections, reasoning, and intelligence in the short term, and it favors children's academic performance in the long term. 42 In line with this statement, in the current study, a significant difference was found in the academic competence of CI-using children ( Table 3 ), who went from a level below the lower average (which indicates the need for intervention in academic performance and the conditions that favor it) to average competence, with results within the average.

In this context, the development of social skills, behavior problems, and academic competence can be significantly correlated with the seven social functions of music proposed by Koelsch. 33 These functions play a central role in shaping children's ability to interact and collaborate effectively in various contexts. Music training, as a social tool, facilitates emotional and cognitive processes that contribute to the regulation of social behavior and interactions. Engaging in music training can improve communication and empathy, for example, which are essential to establish positive peer relationships and enhance cooperation within group settings. Furthermore, music training's ability to synchronize actions and foster social cohesion can lead to improved behavior, reducing disruptive tendencies by promoting shared experiences and mutual understanding. These positive outcomes, in turn, can create a conducive environment for academic success, as children develop the skills necessary to manage emotions, work collaboratively, and remain engaged in learning activities. Thus, incorporating music training into educational and therapeutic interventions may not only address behavioral challenges but also foster academic competence and emotional well-being and quality of life. 10

It is important to highlight that the learning process used in the music training of the current study is defined as a knowledge-building process. Hence, the longer the exposure to structured and sequenced musical activities, the more expressive the results. 38 In the present study, the music training lasted 6 months, with significant changes in most of the factors assessed, which enables us to deduce that there could be a continuity in the evolution of social skills, behavioral problems, and academic competence.

First, one may question whether the improvement in these factors would not be due to the development process expected for the age. This hypothesis is nullified by previous studies 23 29 in which the authors performed structured and sequenced music training with the same duration as in the current study, in children of the same age group, and found significant differences in social skills, behavior problems, and academic competence only in those submitted to music training, whereas the control children presented results without significant differences throughout 6 months. Moreover, differences in these factors started to occur after the beginning of auditory training, as no significant difference was observed between the reference values—M1 (2 months before the beginning of auditory training) and M2 (immediately before its beginning)—with 2 months between them ( Tables 1 2 3 ).

The lack of a control group in the current study is not a weakness when considering the study design. The longitudinal analysis with controlled sample heterogeneity, as individuals controlled themselves, eliminates variables such as quality of stimulation, social level, and so forth, which generally hinders conclusive discussions. However, it is important to note that the teachers were not blinded to the intervention, which can be considered a bias.

The results are promising and lead to further studies that can be conducted with methodological replication and larger samples, enabling for a more in-depth analysis of the data and a broader understanding of the effect of music training as an integrative health practice. 43

## Conclusion

The scientific evidence obtained in the current study demonstrates that CI-using children submitted to music training experienced improvement in their social, behavioral, and academic skills from their schoolteacher's perspective.

## Notes

Appendix 1 Lesson Example **Intervention** : Music Learning Theory (MLT)

**Objectives**
To promote immersion in a variety of tonalities and in binary, ternary, odd irregular, and even irregular meters by activating learning channels through activities that combine body movement, breathing, tonal and rhythmic patterns (acculturation and imitation), harmony, and improvisation.
**Musical material**
Children's songs suitable and adapted for the 6-to-10-year-old age group, specifically composed to emphasize musical aspects (harmonic function, tonality, pulse, rhythmic or tonal pattern) by music educators specializing in MLT. Songs include those with and without lyrics, sung with the neutral syllable “PAM” or recited with the neutral syllable “PA,” performed in a natural a-cappella voice, and occasionally with simple instrumental accompaniment (example: a single instrument like the ukulele).
**Play materials**
Fabrics of various textures and sizes, toys, balls, and soap bubbles to explore movement aspects related to fluidity, weight, and speed. Percussion instruments include claves, egg shakers, castanets, wooden shakers, jingles, bells, metallophones, hand drums, and tambourines. Welcome Activity

1. **Song: “Music class”** (Lydian tonality/duple meter).
  *Material* : None.
  A song to welcome the students—accompanied by the teacher playing the ukulele and singing. The students join with a percussion instrument and sing along.
  All sit in a circle on a mat.
  *Tonal and Rhythmic Musical Activities*
2. **Song: “Little balloon”** (Major tonality/duple meter).
  *Material* : Elastic band.
  Students sit in a circle with the teacher while the teacher sings:
  ○ **Part A** : Perform fluid macropulse movements.
  ○ **Part B** : Perform micropulse movements.
  **Acculturation tonal pattern/Tonal pattern V-I/Emphasize the tonic note of the song.**
3. **Song: “Vestidos de lunares”** (Phrygian tonality/triple meter).
  *Material* : Parachute.
  Students stand holding the parachute. Contrast the song's sections:
  ○ First section: Perform fluid movements.
  ○ Second section: Perform pulse movements.
  Emphasize the tonic note and tonal pattern V-I.
4. **Song: “Owl”** (Lydian tonalit /duple meter).
  *Material* : Satin rings.
  Students sit in a circle with the teacher. They hold satin rings and make fluid pulse movements as the teacher sings.
  On the sound “u,” they raise and lower the fabric.
  **Tonal pattern V-I/Emphasize the tonic note. Students repeat the tonal pattern.**
5. **Song: “Scooter”** (Dorian tonality/duple meter).
  *Material* : None.
  Students and teacher stand. Explore locomotor and stationary movements through the song.
    ○ As the teacher sings, students walk around the room without a set path.
    ○ When the teacher stops singing, students stop walking and emphasize the tonic by singing “PAM.”
  After a few repetitions, the teacher explains that when the singing stops, students will sing “PAM” and interact with a classmate.
  **Emphasize the tonic note and tonal pattern V-I.**
6. **Song: “Dinosaur”** (triple meter).
  *Material* : Drum.
  Students stand while the teacher recites the song. Students walk around the room without a defined direction, exploring body weight.
  ○ They walk to the macropulse and later the micropulse played on the drum by the teacher.
  ○ Students then accompany the song on the drum and with their singing, imitating the teacher.
  **Acculturation rhythmic pattern.**
7. **Song: “Seahorse”** (Aeolian tonality/duple meter).
  *Material* : Helanca fabric.
  Students sit in a circle with the teacher. As the teacher sings, students make wave movements in the micropulse with the fabric.
  After a few repetitions, students switch to wave movements in the macropulse.
  **Emphasize the tonic note/Tonal pattern V-I.**
8. **Song: “Guacamole”** (odd irregular meter).
  *Material* : None.
  Students stand.
  ○ **Part A** : Walk in the micropulse around the room without a set path.
  ○ **Part B** : Stand still and move in the macropulse.
9. **Song: “El baile de las nubes”** (Mixolydian tonality/even irregular meter).
  *Material* : Large voile scarf.
  Students sit in a circle with the teacher. Students play with the scarf during the song by making fluid movements.
  **Emphasize the tonic note/Tonal pattern V-I.**
10. **Song: “The sled”** (Harmonic minor tonality/duple meter).
  *Material* : None.
  Students sit in a circle with the teacher. This activity involves a dance.
  ○ **Part A** : Circular dance.
  ○ **Part B** : Open and close the circle.
  **Acculturation tonal pattern/Tonal pattern V-I/Emphasize the tonic note.**
  *Relaxation*
11. **Song: “Gymnopédie – Satie”** .
  *Material* : Plastic balls.
  All students sit in a circle and use the balls to massage their bodies.
  *Goodbye Activity*
12. **Song: “Bye”** (same as “Music class”).
  *Material* : None.
  A farewell song accompanied by the teacher playing the ukulele and singing.
  Students join with a wooden shaker and sing along.
  All sit in a circle on a mat.

## References

[1] Aksu B Kara H Ataş A Effect of music integrated phonological awareness program on preschool cochlear implant users Int J Pediatr Otorhinolaryngol 2024 180 111923 10.1016/j.ijporl.2024.111923 38636180

[2] Lo C Y Looi V Thompson W F McMahon C M Music training for children with sensorineural hearing loss improves speech-in-noise perception J Speech Lang Hear Res 2020 63 06 1990 2015 10.1044/2020_JSLHR-19-00391 32543961

[3] Nicastri M Lo Castro F Giallini I Inguscio BM S Mariani L Portanova G Vocal singing skills by cochlear implanted children without formal musical training: Familiar versus unfamiliar songs Int J Pediatr Otorhinolaryngol 2023 170 111605 10.1016/j.ijporl.2023.111605 37245390

[4] Persici V Santangelo M Guerzoni L Cuda D Gordon R L Majorano M Music Exposure and Maternal Musicality Predict Vocabulary Development in Children with Cochlear Implants Music Percept 2024 41 04 240 261 10.1525/mp.2024.41.4.240

[5] Ching T Y Is early intervention effective in improving spoken language outcomes of children with congenital hearing loss? Am J Audiol 2015 24 03 345 348 10.1044/2015_AJA-15-0007 26649545 PMC4659415

[6] Good A Gordon K A Papsin B C Nespoli G C Hopyan T Peretz I Russo F A Benefits of music training for perception of emotional speech prosody in deaf children with cochlear implants Ear Hear 2017 38 04 455 464 10.1097/AUD.0000000000000402 28085739 PMC5483983

[7] Hidalgo C Falk S Schön D Speak on time! Effects of a musical rhythmic training on children with hearing loss Hear Res 2017 351 11 18 10.1016/j.heares.2017.05.006 28552493

[8] Gfeller K Driscoll V Schwalje A Beyond technology: The interaction of perceptual accuracy and experiential factors in pediatric music engagement Otol Neurotol 2019 40 03 e290 e297 10.1097/MAO.0000000000002123 30741909 PMC6373483

[9] Looi V Torppa R Prvan T Vickers D The role of music in families of children with hearing loss and normal hearing in Australia, Finland, and the UK Front Neurosci 2019 13 1002 10.3389/fnins.2019.01002 31680796 PMC6798058

[10] Lo C Y Looi V Thompson W F McMahon C M Beyond audition: psychosocial benefits of music training for children with hearing loss Ear Hear 2022 43 01 128 142 10.1097/AUD.0000000000001083 34133401

[11] Yu M Xu M Li X Chen Z Song Y Liu J The shared neural basis of music and language Neuroscience 2017 357 208 219 10.1016/j.neuroscience.2017.06.003 28602921

[12] Dobbs D Reflexos reveladores Mente & Cérebro 2006 161 46 51

[13] Rizzolatti G Sinigaglia C Mirrors in the brain: How our minds share actions and emotions Oxford University Press 2008 10.1093/oso/9780199217984.001.0001

[14] Ferrari P F Coudé G Mirror neurons, embodied emotions, and empathy Academic Press 2018. p.67 77 10.1016/B978-0-12-805397-3.00006-1

[15] Ahmed M F Khater A Tinnitus suppression after cochlear implantation in patients with single-sided deafness Egypt J Otolaryngol 2017 33 61 66 10.4103/1012-5574.199404

[16] Paquette S Rigoulot S Grunewald K Lehmann A Temporal decoding of vocal and musical emotions: Same code, different timecourse? Brain Res 2020 1741 146887 10.1016/j.brainres.2020.146887 32422128

[17] CDaCI Investigative Team Barker D H Quittner A L Fink N E Eisenberg L S Tobey E A Niparko J K Predicting behavior problems in deaf and hearing children: the influences of language, attention, and parent-child communication Dev Psychopathol 2009 21 02 373 392 10.1017/S0954579409000212 19338689 PMC2730756

[18] Gentili N Holwell A Mental health in children with severe hearing impairment Adv Psychiatr Treat 2011 17 01 54 62 10.1192/apt.bp.109.006718

[19] Van Eldik T Mental health problems of Dutch youth with hearing loss as shown on the Youth Self Report Am Ann Deaf 2005 150 01 11 16 10.1353/aad.2005.0024 15969220

[20] Van Grol L DS Andretta I Habilidades Sociais e Variáveis Sociodemográficas em Crianças com Idade Escolar: Um Estudo Descritivo Temas Psicol 2016 24 03 1129 1138 10.9788/TP2016.3-17

[21] Boerrigter M Vermeulen A Marres H Mylanus E Langereis M Frequencies of Behavioral Problems Reported by Parents and Teachers of Hearing-Impaired Children With Cochlear Implants Front Psychol 2019 10 1591 10.3389/fpsyg.2019.01591 31379656 PMC6646424

[22] World Health Organization Life skills education for children and adolescents in schools.Pt. 3, Training workshops for the development and implementation of life skills programmes (No. WHO/MNH/PSF/93.7 B. Rev. 1). World Health Organization;1997

[23] Said P M Abramides D VM Effect of music education on the promotion of school performance in children CoDAS 2020 32 01 e20180144 10.1590/2317-1782/20192018144 32049150

[24] Assis-Fernandes R P Bolsoni-Silva A T Educational social skills and repertoire of children differentiated by behavior and sex. Paidéia (Ribeirão Preto) 2020 30 e3015 10.1590/1982-4327e3015

[25] Deus ACd Fava D C Desenvolvendo civilidade e empatia na infância por meio da música Rev Bras Ter Cogn 2019 15 02 120 125 10.5935/1808-5687.20190017

[26] França-Freitas M LPD Prette A D Del Prette Z A Habilidades sociais e bem-estar subjetivo de crianças dotadas e talentosas Psico-USF 2017 22 01 1 12 10.1590/1413-82712017220101

[27] Casali-Robalinho I G Del Prette Z AP Del Prette A Habilidades sociais como preditoras para problemas de comportamento em escolares Psicol, Teor Pesqui 2015 31 03 321 330 10.1590/0102-37722015032110321330

[28] Gresham F M Elliott S N Inventário de Habilidades sociais, problemas de comportamento e competência acadêmica para crianças: SSRS manual de aplicação, Apuração e Interpretação[DEL PRETTE, ZAP; FREITAS, LC; BANDEIRA, M.; DEL PRETTE, A. autores da adaptação e padronização brasileira].São Paulo Pearson 2016

[29] Said P M Albano D M Abramides D VM Benefícios do treinamento musical na promoção das habilidades sociais e melhora dos problemas de comportamento em crianças Rev Contemp. 2023 3 12 31940 31961 10.56083/RCV3N12-361

[30] Edwards E St Hillaire-Clarke C Frankowski D W Finkelstein R Cheever T Chen W G NIH music-based intervention toolkit: music-based interventions for brain disorders of aging Neurology 2023 100 18 868 878 10.1212/WNL.0000000000206797 36639235 PMC10159759

[31] Boal-Palheiros G Ilari B Music, drama, and social development in Portuguese children Front Psychol 2023 14 1.093832E6 10.3389/fpsyg.2023.1093832 PMC10267308 37325740

[32] Schellenberg E G Corrigall K A Dys S P Malti T Group music training and children's prosocial skills PLoS One 2015 10 10 e0141449 10.1371/journal.pone.0141449 26506414 PMC4624672

[33] Koelsch S Brain correlates of music-evoked emotions Nat Rev Neurosci 2014 15 03 170 180 10.1038/nrn3666 24552785

[34] Torppa R Huotilainen M Why and how music can be used to rehabilitate and develop speech and language skills in hearing-impaired children Hear Res 2019 380 108 122 10.1016/j.heares.2019.06.003 31265971

[35] Shukor N FA Lee J Seo Y J Han W Efficacy of Music Training in Hearing Aid and Cochlear Implant Users: A Systematic Review and Meta-Analysis Clin Exp Otorhinolaryngol 2021 14 01 15 28 10.21053/ceo.2020.00101 32646208 PMC7904420

[36] Innes-Brown H Marozeau J P Storey C M Blamey P J Tone, rhythm, and timbre perception in school-age children using cochlear implants and hearing aids J Am Acad Audiol 2013 24 09 789 806 10.3766/jaaa.24.9.4 24224987

[37] Yucel E Sennaroglu G Belgin E The family oriented musical training for children with cochlear implants: speech and musical perception results of two year follow-up Int J Pediatr Otorhinolaryngol 2009 73 07 1043 1052 10.1016/j.ijporl.2009.04.009 19411117

[38] Gordon E E Teoria de aprendizagem musical para recém-nascidos e crianças em idade pré-escolar Lisboa Fundação Calouste Gulbenkian 2008

[39] Raven J Raven J C Court J H CPM Raven – Matrizes Progressivas Coloridas de Raven – Manual. 1ª ed São Paulo Pearson Clinical Brasil 2018

[40] Gresham F M Elliott S N Social skills rating system: Manual Circle Pines, MN American Guidance Service 1990

[41] Freitas L C Del Prette Z A Social skills rating system-Brazilian version: New exploratory and confirmatory factorial analyses Av Psicol Latinoam 2015 33 01 135 156 10.12804/apl33.01.2015.10

[42] Ishiguro C Ishihara T Morita N Extracurricular music and visual arts activities are related to academic performance improvement in school-aged children NPJ Sci Learn 2023 8 01 7 10.1038/s41539-023-00155-0 36991031 PMC10060367

[43] World Health Organization WHO traditional medicine strategy: 2014–2023 World Health Organization 2013

